# Care or Profit? Unpacking Private Equity’s Impact on U.S. Nursing Homes – A Systematic Review

**DOI:** 10.1093/geroni/igaf122.156

**Published:** 2025-12-31

**Authors:** Gregory Orewa, Aizhan Karabukayeva, Rohit Pradhan, Itopa Jimoh, Robert Weech-Maldonado

**Affiliations:** The University of Texas at San Antonio, San Antonio, Texas, United States; The University of Oklahoma, Norman, Oklahoma, United States; Texas State University, San Marcos, Texas, United States; The University of Alabama at Birmingham, Birmingham, Alabama, United States; The University of Alabama at Birmingham, Birmingham, Alabama, United States

## Abstract

In the past two decades, private equity (PE) acquisitions in nursing homes have surged, driven by financial gain and market consolidation. The increasing presence of PE firms in the U.S. nursing home industry has raised concerns about its impact on care quality and financial performance. This systematic review examined the impact of PE ownership on U.S. nursing homes, focusing on care quality and financial performance using agency theory and the structure-process-outcome framework. Adhering to PRISMA guidelines, a systematic search across five databases, including PubMed, Web of Science, and ABI/Inform. The initial search yielded 343 articles. After removing duplicates, screening for title, abstract, thorough evaluation, and full-text review, 12 studies published between 2000 and 2024 met the inclusion criteria and were included in the study. Data were extracted and synthesized across quality and financial dimensions. PE ownership was linked to higher deficiencies, increased hospitalizations, and higher mortality, though some care processes improved. PE-owned facilities showed initial financial gains but faced long-term financial challenges due to high debt and lease obligations. Staffing changes suggested an increased reliance on lower-skilled staff. PE strategies may prioritize short-term profitability, at the expense of care quality. Cost-cutting measures can undermine staffing and patient outcomes, highlighting the need for stricter staffing regulations, financial transparency, and value-based reimbursement to ensure sustainable, high-quality care. Effective policy interventions are needed to safeguard residents of nursing homes and maintain equitable care standards.

